# Nerve pathology of microangiopathy and thromboinflammation in hereditary transthyretin amyloidosis

**DOI:** 10.1002/acn3.51930

**Published:** 2023-10-30

**Authors:** Shin‐Joe Yeh, Ti‐Yen Yeh, Yi‐Shiang Wang, Chi‐Chao Chao, Shiou‐Ru Tzeng, Tsz‐Yi Tang, Jung‐Hsien Hsieh, Yu‐Yu Kan, Wei‐Kang Yang, Sung‐Tsang Hsieh

**Affiliations:** ^1^ Department of Neurology National Taiwan University Hospital Taipei Taiwan; ^2^ Department of Anatomy and Cell Biology National Taiwan University College of Medicine Taipei Taiwan; ^3^ Institute of Biochemistry and Molecular Biology National Taiwan University College of Medicine Taipei Taiwan; ^4^ Department of Urology Kaohsiung Medical University Hospital, Kaohsiung Medical University Kaohsiung Taiwan; ^5^ Department of Urology Kaohsiung Municipal Siaogang Hospital Kaohsiung Taiwan; ^6^ Department of Surgery National Taiwan University Hospital Taipei Taiwan; ^7^ Department of Anatomy and Cell Biology, School of Medicine College of Medicine, Taipei Medical University Taipei Taiwan; ^8^ School of Medicine, College of Medicine, National Sun Yat‐Sen University Kaohsiung Taiwan; ^9^ Graduate Institute of Clinical Medicine, National Taiwan University College of Medicine Taipei Taiwan; ^10^ Graduate Institute of Brain and Mind Sciences, National Taiwan University College of Medicine Taipei Taiwan; ^11^ Center of Precision Medicine National Taiwan University College of Medicine Taipei Taiwan

## Abstract

**Objective:**

Despite amyloid deposition as a hallmark of hereditary transthyretin amyloidosis (ATTRv) with polyneuropathy, this pathology could not completely account for nerve degeneration. ATTRv patients frequently have vasomotor symptoms, but microangiopathy hypothesis in ATTRv was not systemically clarified.

**Methods:**

This study examined the vascular pathology of sural nerves in ATTRv patients with transthyretin (TTR) mutation of p.Ala117Ser (TTR‐A97S), focusing on morphometry and patterns of molecular expression in relation to nerve degeneration. We further applied human microvascular endothelial cell (HMEC‐1) culture to examine the direct effect of TTR‐A97S protein on endothelial cells.

**Results:**

In ATTRv nerves, there was characteristic microangiopathy compared to controls: increased vessel wall thickness and decreased luminal area; both were correlated with the reduction of myelinated fiber density. Among the components of vascular wall, the area of collagen IV in ATTRv nerves was larger than that of controls. This finding was validated in a cell model of HMEC‐1 culture in which the expression of collagen IV was upregulated after exposure to TTR‐A97S. Apoptosis contributed to the endothelial cell degeneration of microvasculatures in ATTRv endoneurium. ATTRv showed prothrombotic status with intravascular fibrin deposition, which was correlated with (1) increased tissue factor and coagulation factor XIIIA and (2) reduced tissue plasminogen activator. This cascade led to intravascular thrombin deposition, which was colocalized with upregulated p‐selectin and thrombomodulin, accompanied by complement deposition and macrophages infiltration, indicating thromboinflammation in ATTRv.

**Interpretation:**

Microangiopathy with thromboinflammation is characteristic of advanced‐stage ATTRv nerves, which provides an add‐on mechanism and therapeutic target for nerve degeneration.

## Introduction

Hereditary transthyretin amyloidosis (ATTRv) is a progressive multisystem disease due to transthyretin (TTR) mutations, typically affecting motor, sensory, and autonomic nerves.^1^ This disease usually leads to dependent ambulation or death within 3–10 years before the availability of disease‐modifying therapies.^2^, ^3^ The extracellular deposition of amyloid fibrils is a pathology hallmark of ATTRv, mainly in peripheral nerves, heart, and gastrointestinal tissues.^4^, ^5^ In the peripheral nervous system, amyloid deposition is predominantly located in the endoneurium adjacent to microvasculatures and Schwann cells.^6^, ^7^, ^8^ Studies of TTR‐amyloid neurotoxicity mainly focused on neuronal mechanisms, for example, binding of TTR‐amyloid to the receptors of advanced glycation end products followed by oxidative stress and apoptotic death at the level of neuronal cell bodies.^9^

Despite the availability of silencing and stabilizing treatments for ATTRv, some patients continued experiencing the decline of neurological functions, that is, an increase in Neuropathy Impairment Score, the primary end‐point of these clinical trials.^2^, ^10^ Furthermore, the relatively low number and uneven distribution of amyloid deposits in peripheral nerves could not fully explain the diffuse and generalized nerve degeneration in ATTRv.^11^, ^12^ The above clinical observation and discrepancy in neurodegeneration pattern raise the possibility of additional mechanisms contributing to nerve degeneration in ATTRv beyond the amyloid pathology. Frequently, ATTRv patients have cold extremities with pallor skin,^13^, ^14^ implying vasculopathy, which was previously attributed to autonomic neuropathy with deranged vasomotor control of microcirculation.^14^, ^15^ Despite the close contact of microvasculatures with circulating TTR, the effects of variant TTR on endothelial cells received little attention except the disrupted blood–nerve barrier or vascular compression by amyloid deposits.^16^, ^17^, ^18^ Exploring this issue is particularly timely in the era of advanced therapies for ATTRv.^19^

To address these issues, this study aimed to investigate the effects of ATTRv on the microvasculatures, by (1) analyzing human ATTRv sural nerve pathology and (2) conducting experiments in an *in vitro* cell model of vascular endothelial cells exposed to variant TTR proteins.

## Materials and Methods

### Participants

Patients who were diagnosed as ATTRv with polyneuropathy due to TTR mutation between October 2001 and November 2020 were consecutively enrolled in this study. The diagnosis of ATTRv was defined as (1) clinical manifestations of polyneuropathy as progressive four‐limb weakness, sensory disturbance, and autonomic dysfunction; (2) electrophysiological evidence of polyneuropathy with axonal degeneration on nerve conduction studies and quantitative sensory testing; and (3) genetic test for TTR mutations following our previous reports.^20^, ^21^ TTR‐p.Ala117Ser (TTR‐A97S) is the major phenotype (> 90%) in Taiwanese patients according to (1) our previous reports and (2) a large survey of genotypes of worldwide ATTRv.^20^, ^21^, ^22^, ^23^, ^24^ In this study, all ATTRv patients had the genotype of TTR‐A97S. Patients with concomitant underlying diseases that would affect peripheral nerves were excluded, such as diabetes mellitus, chronic kidney disease, syphilis, human immunodeficiency virus infection, thyroid disease, autoimmune disease, malignancy, nutritional deficiency, history of using neurotoxic medications, toxins, or alcohol. The sensory‐motor disability was graded using the Coutinho's staging system, ranging from 0 to 3 stages, with higher stages indicating higher disability.^25^ In addition, vasomotor symptoms of skin defined as cold, pallor, or discoloration on skin were evaluated.^13^, ^14^ The sural nerves of these patients with absent response in nerve conduction study were biopsied for diagnostic purpose. The control group consisted of healthy subjects who underwent nerve graft surgeries, and part of the sural nerve graft was used as control samples. This study was approved by the Research Ethics Committee, and informed consent was obtained from all participants.

### Examinations of sural nerves: Morphometry of nerves and vessels

The biopsied sural nerves were processed as semi‐thin sections and stained with toluidine blue to evaluate myelinated fibers and microvasculatures according to our established protocols.^26^ Images were acquired by differential interference contrast microscopy (DM2500, Leica, Germany) and quantified using Image‐Pro Plus software (Media Cybernetics, Rockville, MD). The number of myelinated fibers was normalized to the area of the defined nerve fascicle as myelinated fiber density (MFD). Myelinated nerve fibers were classified into large‐ and small‐myelinated nerves with a cutoff diameter of 5‐μm. The diameter and luminal area of each vessel were measured after defining the outer border and the luminal border of the vessel, and hence, the vessel wall thickness was defined as the difference of the above two diameters. The percentage of luminal area of each vessel was defined as the luminal area divided by the vessel area.

### Immunostaining

The procedures of immunohistochemistry and immunofluorescence staining and quantification of image signals followed our established protocol.^26^, ^27^ Briefly, sural nerve specimens were cryosectioned to 10 μm‐thickness sections and immunostained with specific antibodies (Supplemental Table S1). Immunofluorescence images were acquired using a confocal microscope (Leica TCS SP8 × STED 3X, Wetzlar, Germany) and quantified using ImageJ software (National Institute of Mental Health, Bethesda, MD).

### Generation of recombinant TTR proteins

The production of recombinant proteins followed our previous protocol.^28^ Briefly, the human TTR‐A97S gene was cloned into the pET21a plasmids, which contained a C‐terminal expression tag with LEHHHHHH. Wild‐type TTR (TTR‐Wt) was generated using the TTR‐A97S‐6His template. Expression plasmids were transformed into the BL21 (DE3) strain of *E. coli* expression system. The transformed *E. coli* were lysed in an EmulsiFlex‐C3 high‐pressure homogenizer (Avestin, Ottawa, Canada). The supernatants were purified with nickel‐affinity column chromatography (GE Healthcare, Boston, MA). Recombinant proteins were eluted and purified by size exclusion chromatography (SEC) in the ÄKTA pure system (GE Healthcare, Boston, MA) of fast protein liquid chromatography (FPLC). The tetrameric fractions from a single peak of the SEC profiles were verified by running 15% SDS‐PAGE. Tetrameric protein concentrations were calculated by detecting A_280_ absorbance using a nanophotometer NP 80 (Implen, Westlake Village, CA) with an extinction coefficient (*ε*) of tetramer = 18450 × 4 = 73800 M^−1^ cm^−1^.

### Endothelial cell culture

Human microvascular endothelial cells (HMEC‐1) were maintained in Medium 200 with low serum growth supplement and 50 U/mL penicillin at 37 °C under a humidified atmosphere of 5% CO_2_, and were cultured with TTR‐Wt or TTR‐A97S proteins (1 μM). The culture medium with and without recombinant TTR proteins was renewed every 2 days. Prior studies have shown that TTR proteins aggregate at acidic conditions (e.g., pH 4.4). At neutral pH, the structures of wild‐type and variant TTR proteins remain tetrameric conformations without detectable monomeric species or amyloid deposits for several weeks.^29^, ^30^, ^31^ Our culture medium was maintained at a neutral pH. Thus, the TTR proteins in the medium were stable as a soluble form. After being exposed to TTR proteins for 1 month, these HMEC‐1 cells were collected for immunoblotting. These culture experiments were repeated for 5 times.

### Immunoblotting

Immunoblotting was used to quantify collagen IV in the cultured endothelial cells following our protocol.^23^ Briefly, the abovementioned HMEC‐1 cells were lysed with radioimmunoprecipitation buffer and protease inhibitors. The supernatant proteins (10 μg per sample) from the lysate were resolved using 8% SDS‐PAGE and transferred onto a PVDF membrane. The membrane was washed and incubated with anti‐collagen IV antibody overnight at 4 °C and visualized with ECL substrate following incubation with HRP‐conjugated antibody at room temperature for 1 h.

### Statistical analysis

Continuous variables were shown as mean ± standard deviation, and the difference between two groups was compared using two‐sample *t*‐test or Mann–Whitney *U* test depending on whether the values followed a Gaussian distribution. Categorical data were compared between two groups using Fisher's exact test. *P* values <0.05 were considered significant.

## Results

### Clinical profiles and quantitative nerve pathology

There were 17 patients (15 [88%] men) with clinically and electrophysiologically defined polyneuropathy caused by TTR A97S mutation. At the time of nerve biopsy, all patients had motor weakness and sensory impairment at four limbs, and sensory‐motor axonal polyneuropathy on nerve conduction study. Autonomic dysfunction was confirmed electrophysiologically with absent sympathetic skin response or reduced heat rate variability of RR‐interval variations in 15 (88%) patients. The onset ages of neurological symptoms were at 60.2 ± 4.3 years and the biopsies were performed at 63.2 ± 3.9 years, with the interval between the onset of symptoms and the time of nerve biopsy was 2.9 ± 1.4 years. In 8 (47%) patients, the initial neurological presentation was numbness or weakness in hands. The Coutinho's stages were at stage 2 in 12 (71%) patients and stage 3 in 5 (29%) patients at the time of nerve biopsy. Vasomotor symptoms of the skin were evident in 10 (59%) patients. The control group consisted of four healthy subjects (2 [50%] men), with the age of 49.0 ± 13.8 years at the time of nerve biopsy. There was no statistical difference in age and sex between the two groups.

**Figure 1:**
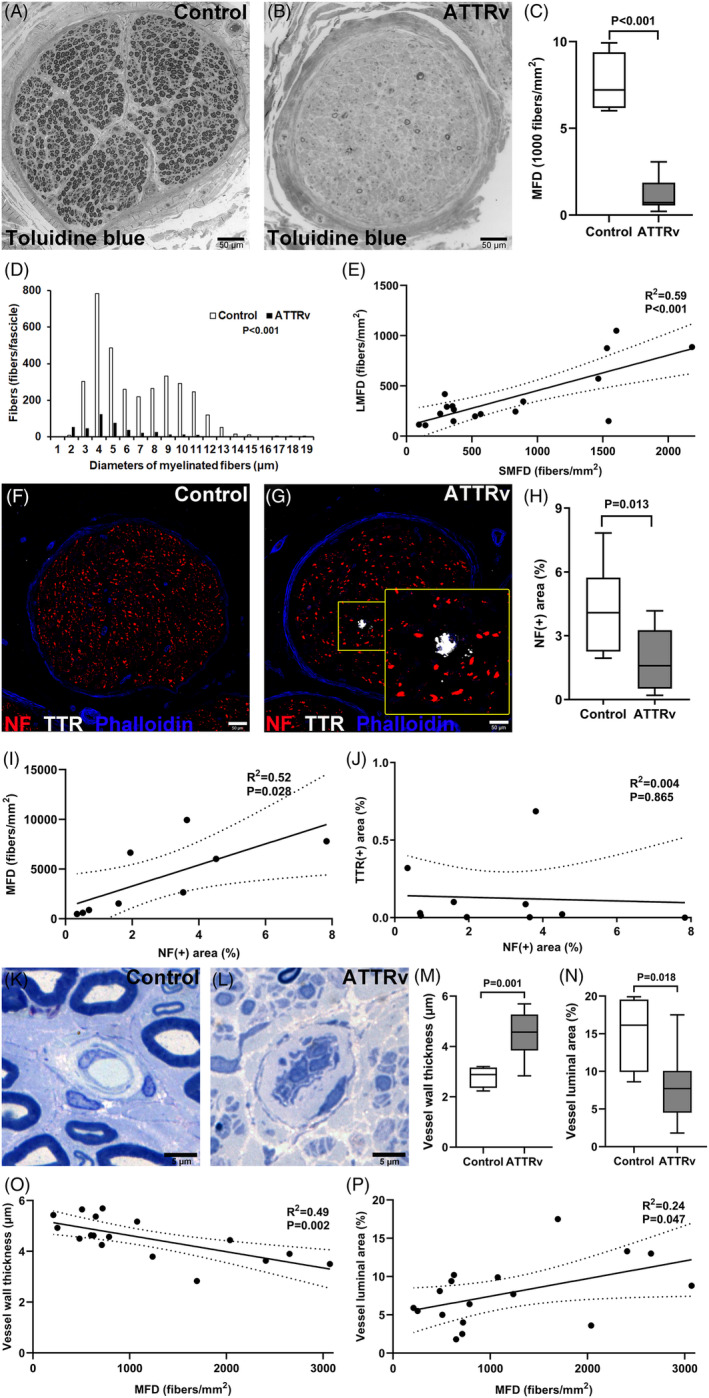
Nerve and vascular pathology of sural nerves in hereditary transthyretin amyloidosis (ATTRv). Sural nerves from ATTRv patients and control subjects were processed for (1) pathology and morphometry of myelinated nerves (A and B) and endoneurial vessels (K and L) on epon‐embedded semi‐thin sections stained with toluidine blue and (2) immunofluorescence staining on cryostat sections (F and G). (A–C) Compared to control nerves (A) with abundant myelinated nerve fibers, there was a lower myelinated fiber density (MFD) in ATTRv nerves (*p* < 0.001, *N* = 17 and 4 in the ATTRv and control groups, respectively) (B and C). Scale bars, 50 μm. (D) Nerve morphometry based on the histogram of myelinated nerve fiber diameters showed absence of a bimodal pattern in ATTRv (*p* < 0.001, *N* = 4 in both groups). (E) Small‐myelinated fiber density (SMFD) was significantly correlated with large‐myelinated fiber density (LMFD) in ATTRv (*p* < 0.001, *N* = 17). (F and G) Immunofluorescence staining for neurofilaments (NF) and transthyretin (TTR) aggregates in the control and ATTRv nerves showed NF(+) area was smaller in ATTRv nerves (H). Scale bars, 50 μm. TTR(+) aggregates were present in some ATTRv nerves (G), with the inset in the right lower corner showing the magnification of the yellow square in (G). (I and J) NF(+) area was correlated with MFD (I), but not with TTR(+) area (J). *N* = 5 and 4 in the ATTRv and control groups in (F–J), respectively. (K–N) On vascular pathology and morphometry, ATTRv nerves had increased thickness of vessel wall (*p* = 0.001) and smaller lumen (*p* = 0.018) compared to controls. Scale bars, 5 μm. (O and P) Both vessel wall thickness (O) and luminal area (P) were correlated with MFD. *N* = 17 and 4 in the ATTRv and control groups in (K–P), respectively.

To understand the patterns of nerve degeneration, we first examined the nerve pathology. Compared to control nerves which contained abundant large‐ and small‐myelinated nerve fibers with normal bimodal distribution, ATTRv nerves had characteristic degeneration pattern on nerve morphometry (Fig. 1A–D), that is, marked reduction of myelinated fiber density with a unimodal distribution on diameter histogram. The significant correlation of the densities between small‐ and large‐myelinated fibers indicated parallel degeneration of both types of nerves (Fig. 1E).

Immunofluorescence staining for neurofilament (NF) and TTR was applied to assess nerve integrity and TTR aggregates, respectively. Compared to controls, there was a reduction of neurofilament(+) areas in ATTRv nerves (Fig. 1F–H). The neurofilament(+) areas were significantly correlated with myelinated fiber density but independent of TTR(+) areas (Fig. 1I,J), implying additional mechanisms for nerve degeneration in ATTRv beyond amyloid deposition.

### Microangiopathy in ATTRv nerves

To investigate the microangiopathy hypothesis, we analyzed microvascular structures in ATTRv nerves. The pattern of microangiopathy was distinct in ATTRv with (1) increased vessel wall thickness (4.5 ± 0.8 μm vs. 2.8 ± 0.4 μm, *p* = 0.001) and (2) smaller luminal area (7.8 ± 4.2% vs. 15.2 ± 5.1%, *p* = 0.018) compared to control nerves (Fig. 1K–N). Furthermore, the myelinated fiber density was (1) inversely correlated with vessel wall thickness (*p* = 0.002) and (2) positively correlated with luminal area in ATTRv (*p* = 0.047) (Fig. 1O,P). The associations of nerve morphometry and microvascular morphometry documented a close relationship between microangiopathy and nerve degeneration, and provided the foundation for following examinations of the downstream cascades, cellular interactions, and molecular mechanisms.

### Increased collagen IV expression with apoptosis in ATTRv microvasculatures

**Figure 2:**
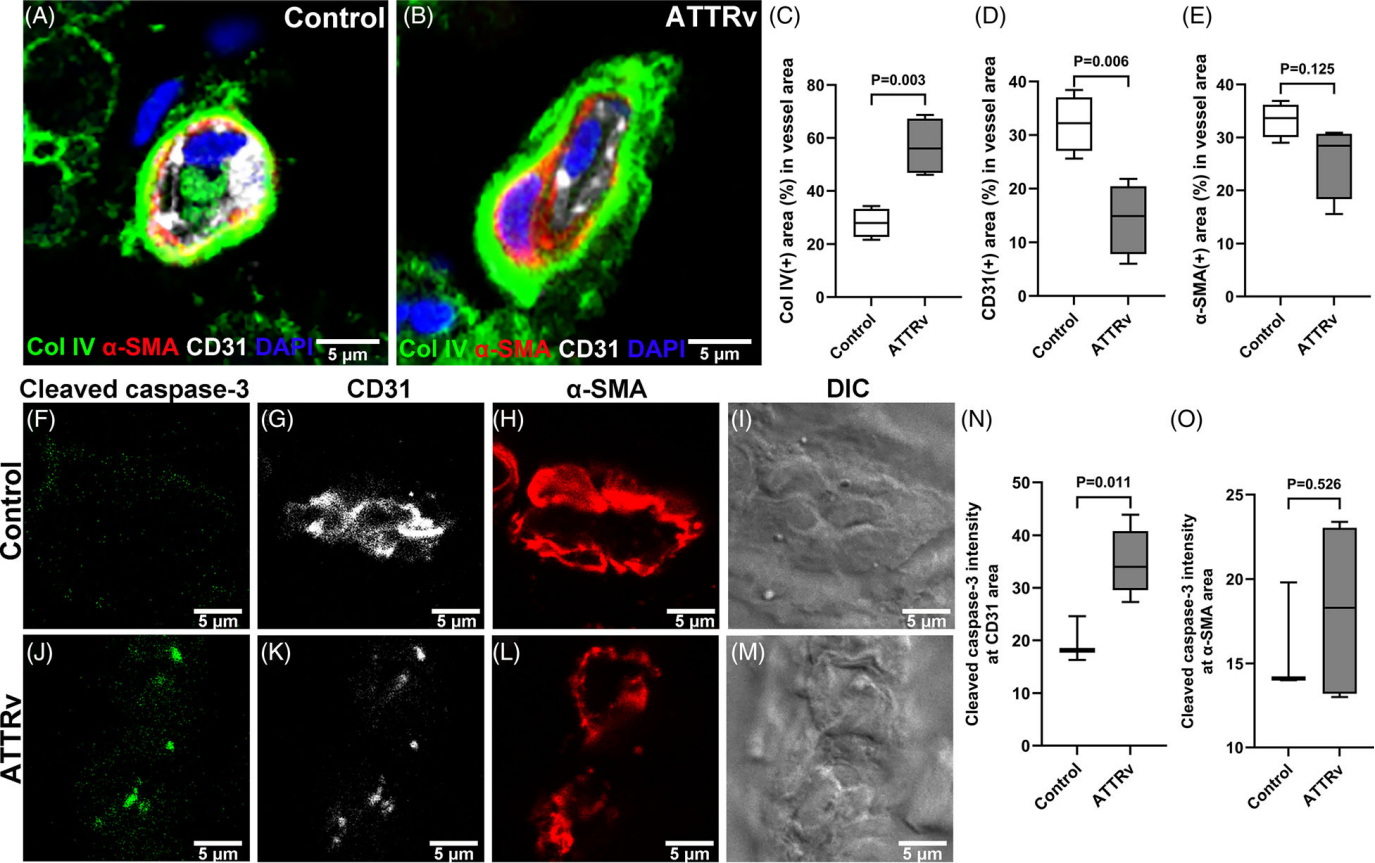
Microangiopathy in the nerves of hereditary transthyretin amyloidosis (ATTRv). (A and B) The immunofluorescence staining of vascular components: endothelial cells (CD31), pericytes (α‐smooth muscle actin, α‐SMA), and basement membrane collagen IV (Col IV) in sural nerves of control (A) and ATTRv (B) showed larger areas of Col IV‐stained basement membrane (*p* = 0.003, C) and smaller areas of CD31‐stained endothelial cells (*p* = 0.006, D) in ATTRv endoneurial vessels, whereas similar areas of pericytes (E) between the two groups. (F–M) The vascular components of endothelial cells (CD31) and pericytes (α‐SMA), and cleaved caspase‐3 were assessed with triple immunofluorescence staining on the nerves from ATTRv (J–M) and control (F–I). Increased expression of cleaved caspase‐3 (*p* = 0.011) was evident in the endothelial cells of ATTRv nerves (N), while there was no significant change in the expression of cleaved caspase‐3 in pericytes (O). *N* = 5 and 3 in the ATTRv and control groups, respectively. Scale bars, 5 μm.

To elucidate the components contributing to the thickened vascular wall of ATTRv, we performed triple immunofluorescence staining with CD31 (for endothelial cells), α‐smooth muscle actin (α‐SMA, for pericytes), and collagen IV (for basement membrane) (Fig. 2A,B). The proportion of collagen IV(+) area in endoneurial vessels was larger in ATTRv than in controls (56.7 ± 10.9% vs. 28.0 ± 5.5%, *p* = 0.003), indicating that collagen IV was responsible for the increased thickness of basement membrane (Fig. 2C). Furthermore, the CD31(+) area was reduced in ATTRv (14.4 ± 6.6% vs. 32.1 ± 5.3%, *p* = 0.006, Fig. 2D). There was no change in α‐SMA, that is, intact pericytes (Fig. 2E).

### Apoptotic endotheliopathy in ATTRv

Given the reduced CD31 expression, we further examined the expression pattern of caspase‐3 to explore the possibility of endothelial cell injury (Fig. 2F–O). In ATTRv nerves, the cleaved caspase‐3 immunoreactivity was evident in endothelial cells (*p* = 0.011, Fig. 2N) but not in pericytes (*p* = 0.526, Fig. 2O). These findings indicated distinct endotheliopathy in ATTRv with apoptosis and vascular wall degeneration corroborating with the reduction of CD31(+) area in endoneurial vessels.

### Increased expression of collagen IV in cultured endothelial cells

**Figure 3:**
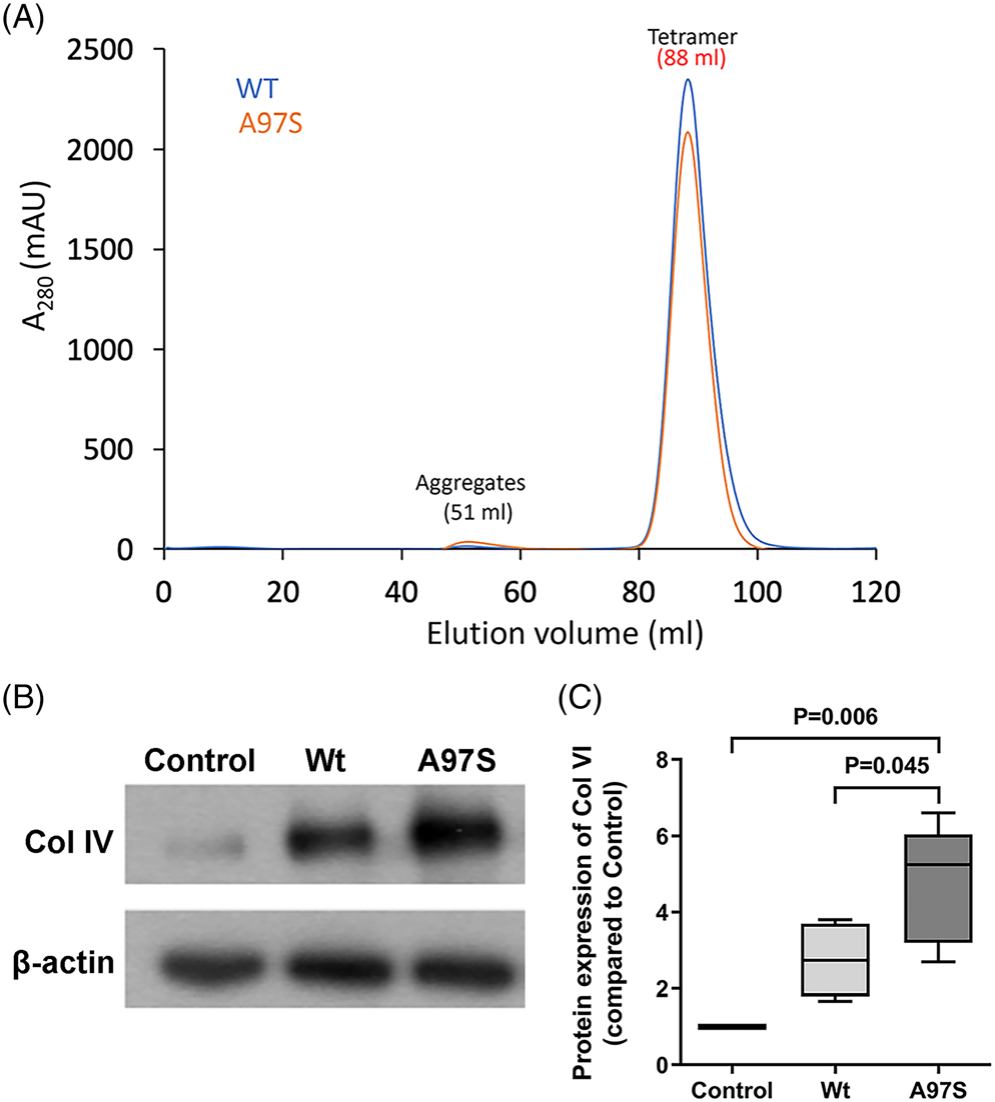
Expression of collagen IV in cultured endothelial cells exposed to transthyretin (TTR) proteins. The purity of recombinant TTR proteins was validated with gel filtration chromatography (A). Then, human microvascular endothelial cells (HMEC‐1) were exposed to recombinant TTR proteins: wild‐type (Wt) vs. variant (A97S) TTR, followed by analyzing collagen IV (Col IV) expression with immunoblotting. (A) Gel filtration chromatography of recombinant TTR proteins. The eluted TTR proteins from Ni‐affinity chromatography were subsequently analyzed using HiLoad Superdex 200 16/600 PG column. The elution profiles of TTR‐Wt were compared with those of TTR‐A97S variants. The tetramer was eluted at 88 mL, while the elution volume of the aggregates was at 51 mL. (B and C) In HMEC‐1 cells, the collagen IV level was increased after an exposure to the variant TTR‐A97S (A97S) protein compared to control (*p* = 0.006) and TTR‐Wt groups (*p* = 0.045). *N* = 5 in each group.

To test the hypothesis that the increased expression of collagen IV was attributed to the variant TTR‐A97S protein, we established an *in vitro* cell model of HMEC‐1 cultured with the medium containing TTR‐Wt or variant TTR‐A97S proteins. We first generated recombinant TTR‐Wt and TTR‐A97S proteins. The purity of the recombinant proteins was confirmed by the single peak at the profiles of fast protein liquid chromatography (Fig. 3A). There was no difference in cell survival between the groups exposed to TTR‐Wt and TTR‐A97S proteins. The expression of collagen IV was significantly higher after an exposure to TTR‐A97S compared to control and TTR‐Wt (*p* = 0.006 and 0.045, respectively; Fig. 3B,C), indicating that TTR‐A97S promoted the production of collagen IV in endothelial cells. This finding provided direct evidence for the influence of TTR‐A97S on endothelial cells, consistent with the *in vivo* data of increased collagen IV expression in the basement membrane of microvasculatures in ATTRv nerves.

### Fibrin deposition in microvasculatures

**Figure 4:**
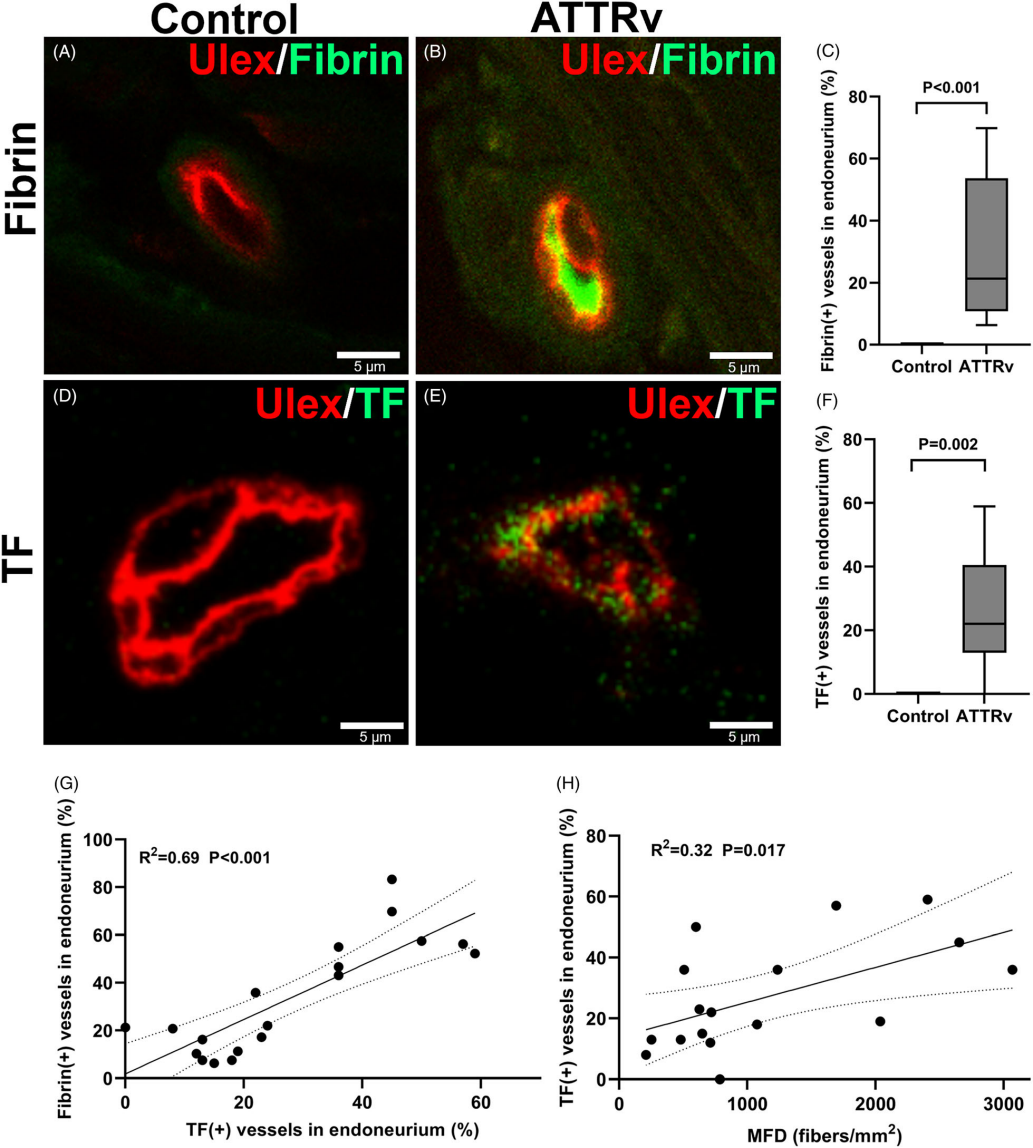
Tissue factor expression with fibrin deposition in microvasculatures of hereditary transthyretin amyloidosis (ATTRv) nerves. The expression of fibrin and tissue factor (TF) in the sural nerves of control and ATTRv subjects was examined by immunofluorescence staining. (A–D) Fibrin deposition was present in the microvasculatures of ATTRv nerves (B) but not in the control nerves (A), with a significant differences in the proportions of fibrin(+) vessels in the endoneurium (*p* < 0.001) (C). (D–F) TF was detected in the vessel wall of ATTRv nerves (E) but not in controls (D), with a significant differences in the proportions of TF(+) vessels in the endoneurium(*p* < 0.001) (F). (G) The proportion of TF(+) vessels was correlated with that of fibrin(+) vessels in the endoneurium of ATTRv nerves (*p* < 0.001). (H) The proportion TF(+) vessels in the endoneurium was correlated with myelinated fiber density (MFD) (*p* = 0.017). *N* = 17 and 4 in the ATTRv and control groups, respectively. Scale bars, 5 μm.

**Figure 5:**
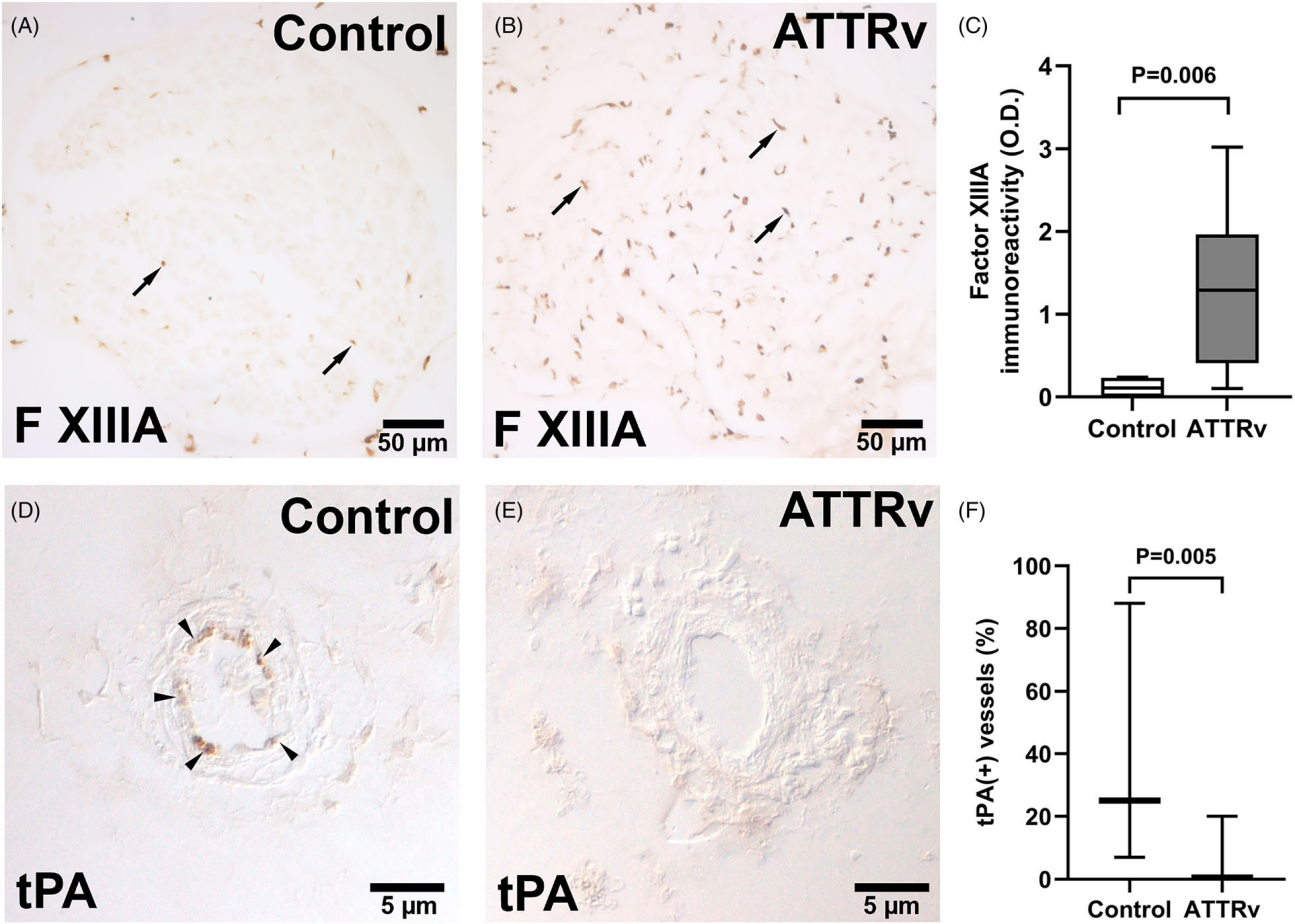
Aberrant thrombosis and thrombolysis activities in the endoneurial microvasculatures of hereditary transthyretin amyloidosis (ATTRv). The expression of factor XIIIA (F XIIIA) and tissue plasminogen activator (tPA) in the sural nerves of the control and ATTRv nerves were immunohistochemically assessed. (A–C) Compared to controls (A), ATTRv nerves (B) had significantly increased expression of factor XIIIA (*p* = 0.006) (C). Arrows in (A) and (B) showed positive staining of factor XIIIA in endoneurial vessels of a nerve fascicle. Scale bars, 50 μm. (D–F) The tPA activity was decreased in the endoneurial vessels of ATTRv compared to controls (*p* = 0.005). Arrowheads in (D) showed positive staining of tPA in endothelial cells of an endoneurial vessel. Scale bars, 5 μm. *N* = 17 and 4 in the ATTRv and control groups, respectively.

To understand whether the coagulation system contributed to the reduced lumen of microvasculatures in ATTRv nerves, we examined the profiles of fibrin‐cascade molecules in endoneurial vessels of nerve biopsies. Fibrin was deposited in the endoneurial vasculatures of ATTRv nerves, but such a phenomenon was not observed in controls (31.5 ± 21.5% vs. 0 ± 0%, *p* < 0.001) (Fig. 4A–C). Since increased fibrin deposition reflected a net outcome between the prothrombotic and thrombolytic systems, we investigated the expressions of the molecules leading to such an imbalance. First, tissue factor, a potent initiator of extrinsic coagulation pathway,^32^ was present only in the microvasculatures of ATTRv nerves (27.2 ± 17.8% vs. 0 ± 0%, *p* = 0.002) (Fig. 4D–F). Furthermore, the proportion of tissue factor(+) vessels in the endoneurium was significantly correlated with (1) the proportion of fibrin(+) vessels (*p* < 0.001, Fig. 4G) and (2) the degree of nerve integrity, that is, myelinated fiber density (*p* = 0.017, Fig. 4H). Second, the expression of coagulation factor XIIIA, which promotes cross‐linking of fibrin clots,^33^ was higher in the microvessels of ATTRv nerves than control nerves (optical density: 1.3 ± 0.9 vs. 0.1 ± 0.1, *p* = 0.006) (Fig. 5A–C). Third, tissue plasminogen activator (tPA), which triggers fibrinolytic pathway,^34^ was lower in the microvasculatures of ATTRv nerves than control nerves (0.04 ± 0.07% vs. 0.40 ± 0.43%, *p* = 0.005) (Fig. 5D–F). Taken together, fibrin deposition in the microvasculatures was attributed to increased expression of prothrombotic molecules and decreased fibrinolytic activities in ATTRv, and the prothrombotic cascade was associated with the severity of nerve degeneration.

### Thromboinflammation in ATTRv nerves

**Figure 6:**
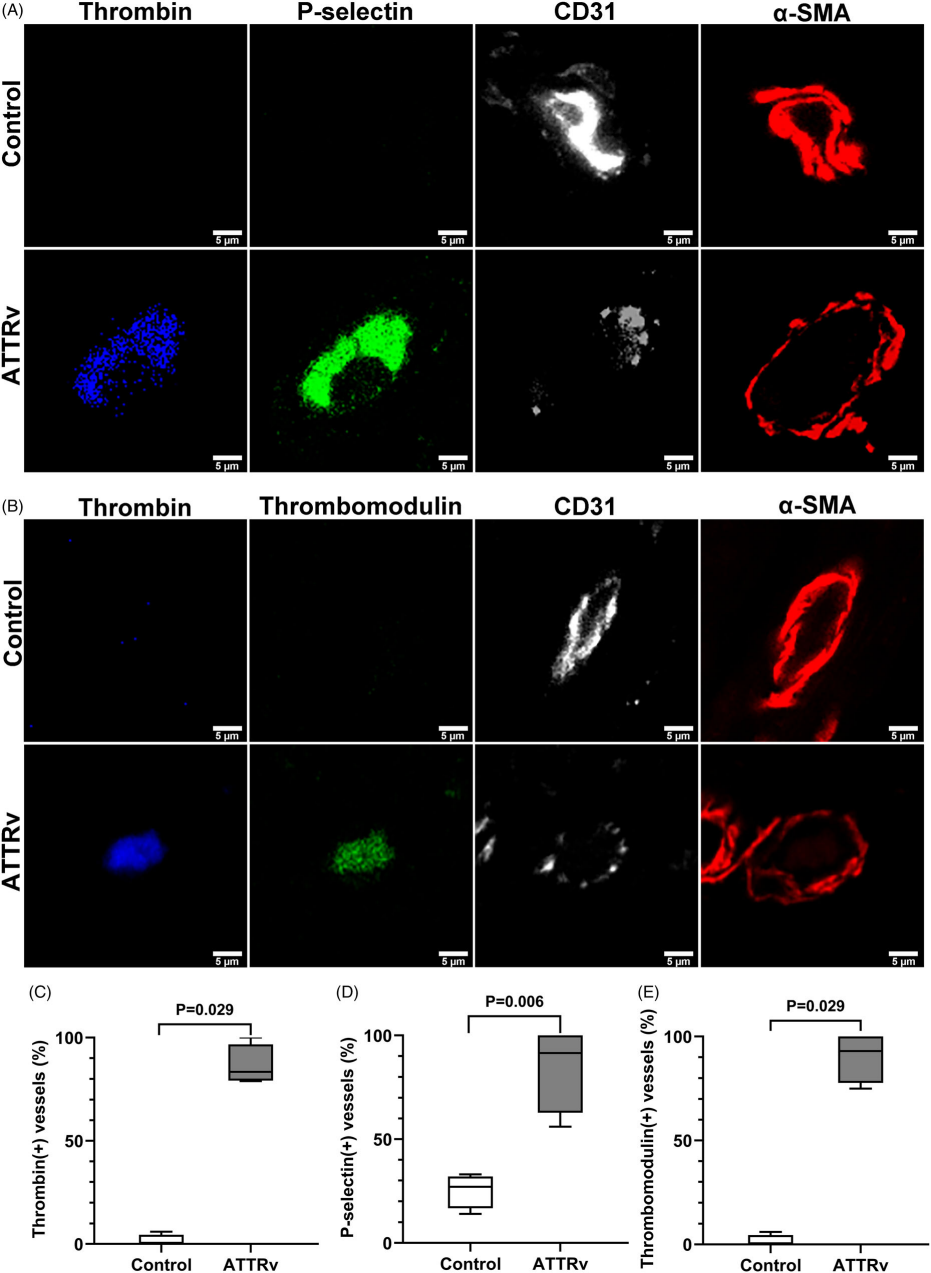
Deposition of thrombin with p‐selectin and thrombomodulin in the endoneurial microvasculatures of hereditary transthyretin amyloidosis (ATTRv). Thrombin, p‐selectin, and thrombomodulin were co‐stained with vascular structural proteins of endothelial cells (CD31) and pericytes (α‐smooth muscle actin, α‐SMA) in the nerves of ATTRv and controls. Endoneurial vessels of ATTRv had increased thrombin deposition (*p* = 0.029; A–C), colocalized with increased expression of p‐selectin (*p* = 0.006; A and D) and thrombomodulin (*p* = 0.029; B and E) compared to controls. *N* = 4 in each group. Scale bars, 5 μm.

To explore the prothrombosis‐induced downstream effects, we further tested the hypothesis of thromboinflammation in ATTRv including platelet activation, complement deposition, and macrophage infiltration.^35^ In endoneurial vessels, multicolor immunofluorescence staining of ATTRv nerves compared to control nerves revealed increased and colocalized expression of thrombin (proportion of thrombin(+) vessels: 86.5 ± 9.7% vs. 1.5 ± 3.0%, *p* = 0.029), p‐selectin (proportion of p‐selectin(+) vessels: 84.8 ± 20.8% vs. 25.3 ± 8.2%, *p* = 0.006), and thrombomodulin (proportion of thrombomodulin(+) vessels: 90.3 ± 12.1% vs. 1.5 ± 3.0%, *p* = 0.029) (Fig. 6), implying the activation of platelets and release of thrombomodulin from endothelial cells in response to thrombin deposition.^36^

**Figure 7:**
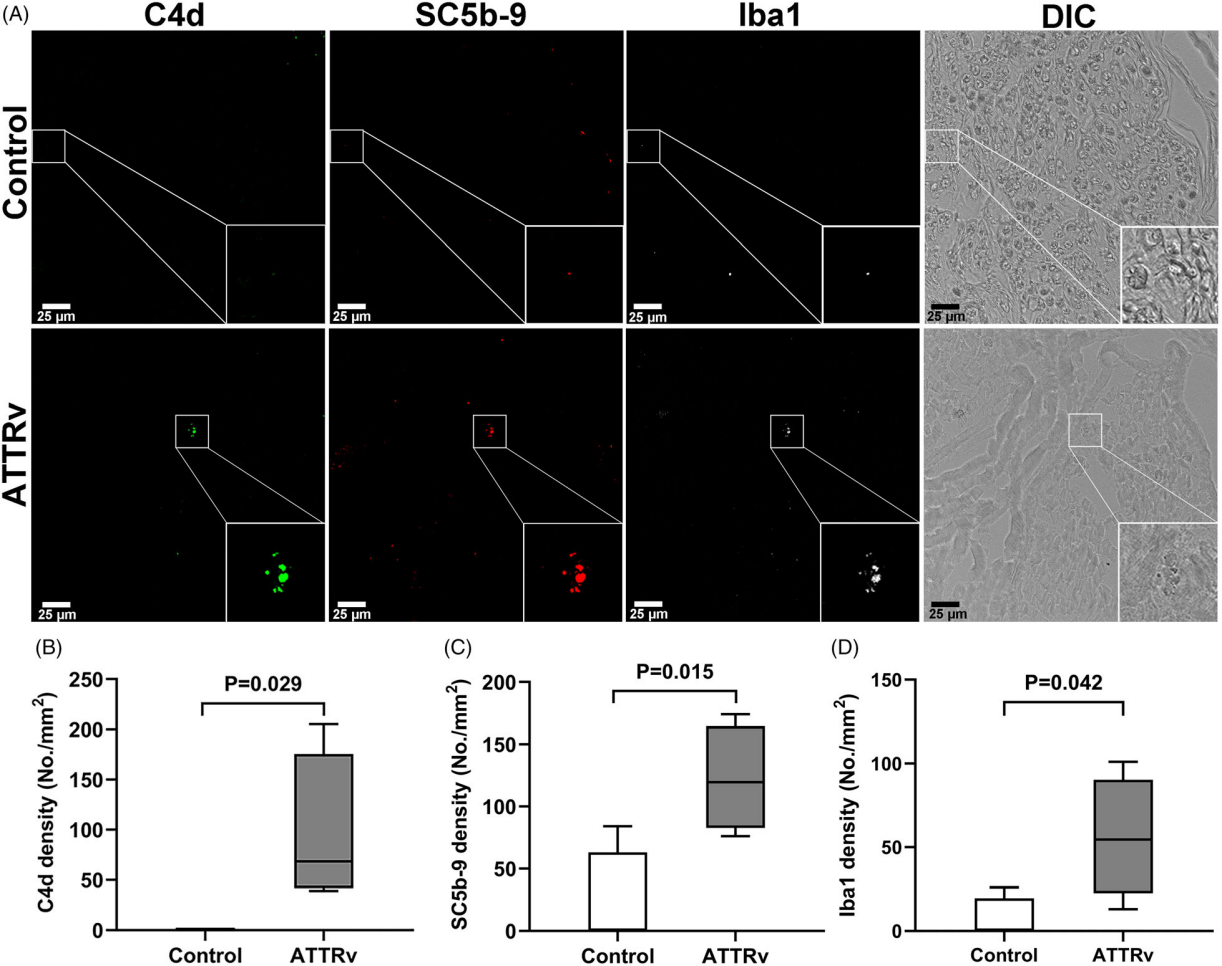
Thromboinflammation in the endoneurial vessels of hereditary transthyretin amyloidosis (ATTRv). Complement proteins (C4d and SC5b‐9) and macrophages (Iba1) were co‐stained in the nerves of ATTRv and controls. (A) C4d and SC5b‐9 were distributed in the endoneurial vessels of ATTRv, and Iba1 signals were colocalized with these two complement proteins. In contrast, the expressions of complement proteins and Iba1(+) macrophages were negligible in controls (B–D). The inset of the right lower corner of each figure in (A) was a magnification of the small square in each figure. *N* = 4 in each group. Scale bars, 25 μm.

The next combination of multiple immunofluorescence staining on endoneurial vessels of ATTRv nerves in comparison with control nerves showed (1) increased deposition of complements, C4d (95.2 ± 75.8 deposits/mm^2^ vs. 0 ± 0 deposits/mm^2^, *p* = 0.029) and SC5b‐9 (122.4 ± 42.5 deposits/mm^2^ vs. 20.9 ± 41.8 deposits/mm^2^, *p* = 0.015), and (2) increased Iba1(+) macrophages (55.8 ± 36.1 counts/mm^2^ vs. 6.5 ± 13.0 counts/mm^2^, *p* = 0.042), indicating complement activation and macrophage infiltration (Fig. 7). Furthermore, Iba1 signals were colocalized with complements inside the vessels of ATTRv. Taken together, these findings provide evidence for thromboinflammation as important pathology hallmark in ATTRv nerves.

## Discussion

This study documented microangiopathy with enhanced thrombotic cascades and thromboinflammation in ATTRv nerves: (1) increased collagen IV expression contributing to thickened basement membrane of endoneurial microvasculatures; (2) apoptotic endotheliopathy and enhanced thrombosis leading to reduced vascular lumen; and (3) thrombus‐induced platelet activation, complement deposition, and macrophage infiltration. These microvascular pathologies contributed to the nerve degeneration in ATTRv. Taken together with significant correlations between microangiopathy parameters (vessel wall thickness and luminal area) and myelinated fiber density, these findings provide *in vivo* and *in vitro* evidence for microangiopathy as a major contributor underlying nerve degeneration in ATTRv.

Previous pathology studies have implied additional mechanisms underlying nerve degeneration in ATTRv beyond amyloid deposition; for example, the pattern of nerve degeneration in ATTRv is generalized,^37^ in contrast to (1) focal amyloid deposition only adjacent to some vessels of sural nerves and (2) low frequency of amyloid deposition in sural nerves compared to other nerves.^11^, ^12^ The absence of correlation between neurofilament(+) nerve fiber area and TTR(+) amyloid area in this study prompted us to test the vascular hypothesis, that is, microangiopathy might serve as an add‐on mechanism contributing to nerve degeneration in ATTRv. Previously, ATTRv vascular studies mainly focused on the deposition of amyloid fibrils and the relationship with the basement membrane of microvasculaures.^37^, ^38^, ^39^ The symptoms of chronic peripheral vascular insufficiency presented as cold and pallor distal extremities were evident in 59% of our patients and in the literature of ATTRv,^16^ mimicking chronic microvascular ischemia in Raynaud's phenomenon.^40^ However, there was a lack of systematic studies to examine the pathology of microangiopathy in peripheral nerves of ATTRv except disruption of blood–nerve barrier.^16^, ^41^ This study provides evidence linking microangiopathy and nerve degeneration in ATTRv: Nerve morphometry (myelinated fiber density) was linearly correlated with (1) vascular wall thickness and (2) luminal area. Increased expression of collagen IV in the basement membrane was a major contributor to the thickened vascular wall in ATTRv nerves, according to our *in vivo* sural nerve pathology and *in vitro* experiments of endothelial cells exposed to the variant TTR‐A97S protein. Furthermore, this study provides evidence that degeneration and apoptosis of endothelial cells with thromboinflammation played important roles in the pathogenesis of microangiopathy in ATTRv. In addition to A97S mutation reported in the present study, microangiopathy in peripheral nerves was also documented in patients with ATTRv carrying V30M or V32G mutations; however, intravascular fibrin deposition was not described before.^3^, ^42^ Beyond peripheral nerves, growing evidence has shown that double‐barreling vessel wall in the leptomeninges was a frequent phenomenon in early stage of ATTRv amyloidosis, which could present as a transient focal neurological deficit, stroke, or cognitive decline, including patients with V30M or non‐V30M mutations.^39^, ^43^ Transient focal neurological deficit was the most common presentation of ATTRv in the central nervous system, occurring in 12–31% of patients.^39^ Furthermore, retinal angiopathy was observed in patients with ATTRv, including tortuosity and obliteration of retinal vessels, leading to irreversible vision loss.^44^ Thus, these observations support the notion that microangiopathy in ATTRv was not confined to peripheral nerves. Ongoing RNA interference or antisense therapies will help to elucidate whether microangiopathy could be reversed and hence nerve degeneration.^2^, ^10^, ^45^

This nerve pathology study further documented a unique observation linking microangiopathy and neuroinflammation, that is, ATTRv nerves are in a procoagulation and prothrombotic status. This observation of prothrombosis in ATTRv nerves at the tissue level was supported by the clinical observation that patients with ATTRv had a higher incidence of early hepatic artery thrombosis after liver transplantation,^46^ and platelet aggregation could be induced by misfolded proteins of amyloid fibrils.^47^ Furthermore, the enhanced thrombotic process and suppressed fibrinolytic activity in the microvasculatures at nerve tissue level of ATTRv merit clinical consideration. So far, advanced silencing or stabilizer therapies focused on TTR itself.^48^ Although the deterioration of neurological deficits was slowed in the clinical trials, some patients continued declining,^2^, ^49^ suggesting that the consequences of remaining variant TTR proteins on endothelial cells and the downstream cascades are likely an add‐on therapeutic target. Certainly, this hypothesis requires further clinical trials and follow‐up studies.

We further demonstrated thromboinflammation following enhanced thrombosis in ATTRv: increased expression of p‐selectin colocalized with thrombin deposition. P‐selectin is expressed on activated platelets and endothelial cells, playing a central role in the development of thrombosis through mediating platelet‐leukocyte and platelet–platelet aggregates and upregulating tissue factor.^50^ Thrombomodulin is released from activated endothelial cells into the circulation, which binds to thrombin to inhibit its procoagulant function.^51^ Activated platelets release complements, further inducing platelet activation and aggregation.^52^ These activated complements affect the functions of macrophages by binding with them,^53^ providing a foundation for our findings of activated complements colocalized with macrophages in endoneurial microvasculatures of ATTRv. Moreover, recent evidence has shown that monocytes are activated to have prothrombotic and proinflammatory features once binding with platelets in inflammatory and autoimmune disorders. Thus, monocyte‐platelet interactions serve as a key element connecting thrombosis and inflammation.^54^ Taken together, thromboinflammation is likely to contribute to and exacerbate the microangiopathy and nerve degeneration of ATTRv.

Many cardinal features of microangiopathy in ATTRv nerves were in concordance with those in diabetic nerves, including increased basement membrane thickness and reduced luminal area of the microvasculatures, with intravascular fibrin deposition and infiltration of macrophages.^26^ Diabetes mellitus is notorious for microvascular complications in major organs of the kidney and retina, and the microangiopathy in peripheral nerves contributes to the complex mechanisms of nerve degeneration.^55^ In contrast, previous studies showed that ATTRv neuropathy was attributed to neurotoxicity of amyloid deposits, whereas the component of microangiopathy received little attention except for disrupted blood–nerve barrier and compression by amyloid deposits.^16^, ^17^, ^18^ Recent evidence revealed that patients with ATTRv had increased proinflammatory cytokines in serum, also in asymptomatic carriers,^56^ indicating the role of inflammation in the early stage of ATTRv probably before amyloid deposition. All patients in this study were free from diabetes. Since the sampled nerves in this study came from advanced stage of ATTRv, the possibility of end‐stage microangiopathy could not be completely excluded and is a limitation of this report. Future studies on sural nerves of ATTRv from early stage ATTRv will answer the issue of specificity.^57^ Nevertheless, the observation of microangiopathy and thromboinflammation provides a new therapeutic target for ATTRv.

This study had limitations. First, the number of control cases was low due to difficulty in obtaining sural nerves from healthy subjects. Second, these ATTRv patients had the same genotype of TTR‐A97S, the most common one in Taiwan and the most frequent non‐TTR p.Val50Met (TTR‐V30M) genotype next to TTR p.Set97Tyr (TTR‐S77T) according to the survey of global genotypes.^20^, ^21^, ^22^, ^23^, ^24^ It is necessary to examine the nerve pathology of other genotypes. Nevertheless, the international collation indicated that the natural course of TTR‐A97S was similar to that of the late‐onset TTR‐V30M.^8^ Thus, the current study paves the way for cross‐genotype comparison.

## Conclusions

This study provides direct evidence of microangiopathy in ATTRv nerves, including increased thickness of vessel wall attributed to upregulated expression of collagen IV responsible for the thickening of basement membrane, apoptosis and degeneration of endothelial cells, and reduced vascular lumen due to thrombus formation with induction of thromboinflammation, providing an add‐on mechanism and therapeutic target for nerve degeneration in ATTRv.

## Author Contributions

Shin‐Joe Yeh, Ti‐Yen Yeh, Tsz‐Yi Tang, and Sung‐Tsang Hsieh contributed to the study conception and design. Material preparation, data collection, and analysis were performed by Shin‐Joe Yeh, Ti‐Yen Yeh, Yi‐Shiang Wang, Tsz‐Yi Tang, Jung‐Hsien Hsieh, Yu‐Yu Kan, and Wei‐Kang Yang. The first draft of the manuscript was written by Shin‐Joe Yeh, and all authors commented on previous versions of the manuscript. All authors read and approved the final manuscript.

## Conflict of Interest

The authors report no conflict of interest.

## Supporting information


Table S1

## Data Availability

The data of this study are available from the corresponding author upon reasonable request.
