# Supplementary material for: Nerve pathology of microangiopathy and thromboinflammation in hereditary transthyretin amyloidosis
Source: Ann Clin Transl Neurol. 2023 Oct 30;11(1):30–44. doi: 10.1002/acn3.51930 (PMC10791016; doi:10.1002/acn3.51930)
Supplement: Supplementary file 1 — Table S1 [file ACN3-11-30-s001.docx]

**Supplemental Table 1. List of primary antibodies**

| Antibody target | Catalog No. | Dilution | Monoclonal/polyclonal | Company |
| --- | --- | --- | --- | --- |
| Tissue factor | AMAb91236 | 1:500 | Monoclonal | Atlas Antibodies, Bromma, Sweden |
| Factor XIIIA | 0102770 | 1:500 | Polyclonal | Origene, Rockville, MD |
| Tissue plasminogen activator | 387 | 1:200 | Polyclonal | American Diagnostica Inc., Stamford, CT |
| Fibrin, coupled to ﬂuorescein isothiocyanate | F0111-1 | 1:200 | Polyclonal | Dako, Santa Clara, CA |
| Rhodamine labeled Ulex Europaeus Agglutinin I | RL-1062 | 1:50 | - | Vector, Newark, CA |
| Collagen IV (immunofluorescence) | 2150-0140 | 1:50 | Polyclonal | Bio-Rad, Hercules, CA |
| Collagen IV (immunoblotting) | ab6586 | 1:1000 | Polyclonal | Abcam, Cambridge, UK |
| α-smooth muscle actin | A5228 | 1:1000 | Monoclonal | Sigma-Aldrich, St. Louis, MO |
| CD31 | ab215912 | 1:50 | Monoclonal | Abcam, Cambridge, UK |
| Cleaved caspase 3 | 9661 | 1:400 | Polyclonal | Cell Signaling, St. Louis, MO |
| C4d | BI-RC4D | 1:200 | Polyclonal | BIOMEDICA DIAGNOSTICS, Windsor, Canada |
| SC5b-9 | A239 | 1:500 | Monoclonal | Quidel, San Diego, CA |
| Iba1 | ab5076 | 1:100 | Polyclonal | Abcam, Cambridge, UK |
| Thrombin | 05-880 | 1:100 | Monoclonal | Merck Millipore, Burlington, MA |
| P-selectin | 84298 | 1:500 | Monoclonal | Cell Signaling, St. Louis, MO |
| Thrombomodulin | PA5-21924 | 1:500 | Polyclonal | Thermo Fisher Scientific, Waltham, MA |
